# *Brahmarasayana* protects against Ethyl methanesulfonate or Methyl methanesulfonate induced chromosomal aberrations in mouse bone marrow cells

**DOI:** 10.1186/1472-6882-12-113

**Published:** 2012-08-01

**Authors:** Kanive Parashiva Guruprasad, Advait Subramanian, Vikram Jeet Singh, Raghavendra Sudheer Kumar Sharma, Puthiya Mundyat Gopinath, Vikash Sewram, Panniyampilly Madhavankutty Varier, Kapaettu Satyamoorthy

**Affiliations:** 1 Division of Biotechnology, Manipal Life Sciences Centre, Manipal University, Planetarium, Complex, Manipal, Karnataka 576 104, India; 2 Oncology Research Unit, South African Medical Research Council, Overport, South Africa; 3 Arya Vaidya Sala, Kottakkal, Kerala 676 503, India; 4 Manipal Life Sciences Centre, Manipal University, Manipal – 576 104, Udupi District, Karnataka, India

**Keywords:** DNA damage and repair, Anti clastogenicity, Rasayana

## Abstract

**Background:**

Ayurveda, the traditional Indian system of medicine has given great emphasis to the promotion of health. Rasayana is one of the eight branches of Ayurveda which refers to rejuvenant therapy. It has been reported that rasayanas have immuno-modulatory, antioxidant and antitumor functions, however, the genotoxic potential and modulation of DNA repair of many rasayanas have not been evaluated.

**Methods:**

The present study assessed the role of Brahmarasayana (BR) on Ethyl methanesulfonate (EMS)-and Methyl methanesulfonate (MMS)-induced genotoxicity and DNA repair in *in vivo* mouse test system. The mice were orally fed with BR (5 g or 8 mg / day) for two months and 24 h later EMS or MMS was given intraperitoneally. The genotoxicity was analyzed by chromosomal aberrations, sperm count, and sperm abnormalities.

**Results:**

The results have revealed that BR did not induce significant chromosomal aberrations when compared to that of the control animals (p >0.05). On the other hand, the frequencies of chromosomal aberrations induced by EMS (240 mg / kg body weight) or MMS (125 mg / kg body weight) were significantly higher (p<0.05) to that of the control group. The treatment of BR for 60 days and single dose of EMS or MMS on day 61, resulted in significant (p <0.05) reduction in the frequency of chromosomal aberrations in comparison to EMS or MMS treatment alone, indicating a protective effect of BR. Constitutive base excision repair capacity was also increased in BR treated animals.

**Conclusion:**

The effect of BR, as it relates to antioxidant activity was not evident in liver tissue however rasayana treatment was observed to increase constitutive DNA base excision repair and reduce clastogenicity. Whilst, the molecular mechanisms of such repair need further exploration, this is the first report to demonstrate these effects and provides further evidence for the role of brahmarasayana in the possible improvement of quality of life.

## Background

It is well known that traditional systems of medicines are playing an important role in global health care needs. India is one of the most medico-culturally diverse countries in the world where Ayurveda, Yoga, Unani, Siddha and other forms of traditional practice have been the mainstay to maintain human health and prevent diseases until the era of modern medicine. In addition to these systems, there are large numbers of folklore healers in this stream who have not been structured under these categories. Among these traditional medicinal systems, Ayurveda is the holistic, comprehensive ancient medical science practiced in India since the 12^th^ Century B.C.

The Ayurvedic therapeutic modality comprises eight angas or chikitsas, each defining a specific branch of study. Rasayana *chikista* (also known as *Jarachikitsa*) encompasses the treatment of ageing through rejuvenation. The enhancement of *Rasa* (essence) is the quintessential quality that all Rasayana medicines possess, ultimately helping to promote health and vigour of the tissues in the body [1]. They have a characteristic tendency to improve the nutritional status of the body and alleviate symptoms of stress through three basic mechanisms - *Rasa* (nutrient effect), *Agni* (digestion and metabolism) and *Srotas* (microcirculation and tissue perfusion) [2]. All Rasayanas are unique formulations of a number of plants, herbs and spices. There are certain organ and tissue specific Rasayanas such as *Medhya* rasayana for the brain, *Hridya* rasayana for the heart, *Twachya* rasayana for the skin and *Chakshusya* rasayana for the eyes. Similarly, they may be age specific as they promote nutrition relevant to natural bio-losses occurring at different phases of human life [2]. Rasayana drugs are rich in antioxidants and are good hepato-protective [1] and immunomodulating [3] agents.

*Brahmarasayana* is one such *Medhya* rasayana comprising of more than 35 ingredients of which the plants *Emblica officinalis* (Goose berry) and *Terminalia chebula* (Indian Gall nut) are the two major. It is reported to retard brain ageing and help in regeneration of neural tissues with anti stress, adaptogenic and memory enhancing effects [2]. BR has often shown to be a potent antioxidant and free radical scavenger in mice [4], heat stressed chicken [5] and cold stressed chicken [6]. BR has also been investigated as an anti-cancer agent, reducing the mass and incidence of palpable tumours in adult malignant rat prostrate tumor cell (MAT-LyLu) inoculated Copenhagen rats [7] and repressing the production of pro-angiogenic factors like vascular endothelial growth factor (VEGF), matrix metalloproteinase 9 (MMP-9) and matrix metalloproteinase 2 (MMP-2) [8]. It has been also observed that BR does not elicit any genotoxic effects. Instead it was shown to increase the mitotic cellularity and altogether alleviate certain types of normally occurring sperm abnormalities [9].

It is well known that the genome of all the organisms including human is susceptible to damages caused by vast array of both endogenous and exogenous DNA damaging agents. Repair of such DNA damages play an important role in cellular functions. Defects in DNA repair or inefficiency of DNA repair machinery results in accumulation of DNA damages in the genome which in turn are responsible for various disorders including cancer and ageing. Although various beneficial effects of Brahmarasayana were investigated, there are no reports to date of BR on potentiation or protection of DNA repair. Hence, the present study was undertaken to evaluate the effect of BR on chromosomal aberrations induced by ethyl methanesulfonate (EMS) and methyl methanesulfonate (MMS), two well-known mutagenic and clastogenic agents in *in vivo* mouse test system. Furthermore, the effect of BR on constitutive base excision repair has also been undertaken to understand its role as a repair enhancer.

## Methods

### Animals

Inbred male Swiss albino mice (*Mus musculus*), 6–8 weeks old and weighing about 30–40 g were used in all experiments. Animals were obtained from the Departmental Animal House. They were maintained in polypropylene shoebox cages with a grill top. Paddy husk was used as bedding material and the animals were fed with standard diet and water *ad libitum*. Three animals were housed per cage and utmost care was taken to maintain cage hygiene and also provide good ventilation and aeration in the room where the animals were housed. The study was approved by the Institutional animal ethics committee (Kasturba Medical College, Manipal) and all experiments were performed in accordance with the guidelines of the ethics committee. Bone marrow cytogenetic assay was employed to determine any clastogenic effects in dividing cells.

### Chemicals

*Brahmarasayana *a commercially available product prepared by Arya Vaidya Sala (Kotakkal, India) was purchased from local Ayurvedic vendors. The manufacture and expiry date of each batch (Batch Nos. 501003, 503232, 503539, 503772 and 504015) was noted. In addition, the HPTLC profile of batch specific product was obtained from the manufacturer and placed on record for authentication and comparison purposes. These data are available upon request. Ethyl methanesulfonate (EMS) (CAS No. 62-50-0) and Methyl methanesulfonate (MMS) (CAS No. 66-27-3) served as standard clastogens and were purchased from Sigma Aldrich (St. Louis, USA).

### Preparation and administration of Brahmarasayana

Brahmarasayana was administered in two different doses. The dose, based on body surface area of mice, was prepared in the form of food pellets, wherein 5 g of BR was mixed with 5 g of animal feed (henceforth known as *Rasayana1*). This mixture was then pressed into pellets which, upon drying for a few hours, became hard and was ideal for chewing. The second dose, based on a body weight of 250 mg/kg body weight/day, was prepared by mixing approximately 8 mg of BR with sterile water (0.5 mL) and administering by gavage (henceforth known as *Rasayana 2*). Two different groups of animals were fed daily for 60 days with either *Rasayana 1* or *Rasayana 2.*

### EMS and MMS administration

Sub-lethal high doses of EMS (240 mg/kg body weight) and MMS (125 mg/kg body weight) were employed (one day after rasayana treatment), as earlier established by Riaz Mahmood and Vasudev [10]. The required volumes of EMS and MMS were dissolved in 0.9% NaCl and the solution (0.5 mL) administered intraperitoneally (i.p.).

### Treatment schedule

#### Control

Mice were provided with regular food. On day 61, these animals received 0.9% NaCl solution (0.5 mL) as intraperitoneal (i.p.) injection.

#### Rasayana

Two groups of animals were fed BR at the level of 5 g /day (*Rasayana 1*: n = 9) and 8 mg /day (*Rasayana 2*: n = 9) for 60 days. On day 61, these animals received 0.9% NaCl solution (0.5 mL) by i.p. injection.

#### Clastogen treatment

Animals were divided into two groups and maintained for 60 days with regular food. On day 61, one of the groups (n = 9) were injected with EMS (240 mg/kg body weight) whilst the other group (n = 9) with MMS (125 mg/kg body weight).

#### Rasayana and EMS-or MMS-combined treatment

Two groups of animals (n = 9 for each group) were fed with *Rasayana 1* (Group 1) and *Rasayana 2* (Group 2) for 60 days. After 60 days, one group of animals belonging to *Rasayana 1* and *Rasayana 2* were injected with EMS and the other group with MMS at the same dose as injected for the clastogen treatment group alone.

### Mitotic preparation and chromosome analysis

Animals were sacrificed at 24 h, 48 h and 72 h after the treatment of clastogen or 0.9% NaCl by i.p. injection. Colchicine (0.05% : 0.5 mL), a spindle fibre inhibitor that arrests cells at metaphase was also administered by i.p. injection 90 minutes prior to sacrifice of these animals. These animals were sacrificed by cervical dislocation and bone marrow cytogenetic assay was employed to determine any clastogenic effects in dividing cells. The bone marrow was processed and slides were prepared by routine standard air dry technique [11]. In brief, the femur bones were dissected and flushed with 0.56% KCl (5 mL) with the help of a 26 gauge needle attached to a syringe (5 mL). The suspension was then incubated at 37°C for 15 to 20 minutes. After incubation, the tubes were centrifuged at 3000 rpm for 10 minutes at 4°C. The supernatant was discarded and cold Carnoy’s fixative (3:1 v/v of methanol: acetic acid, 5 mL) was added and mixed intensively to avoid clumping of cells. The tubes were kept at room temperature for 30 minutes and once again centrifuged for another 10 minutes at 4°C (3000 rpm) and the supernatant removed. This process was repeated twice over 20 minute intervals. Finally the pellet was resuspended in Carnoy’s fixative (0.5 mL) and dropped from a height on a non-greasy chilled glass slide using a fine tipped pasteur pipette. The slides were air-dried, coded and stained with 4% Giemsa for 20–30 minutes. The stained slides were rinsed thoroughly with running tap water followed by distilled water and dried before being viewed under the microscope. Chromosomal aberrations such as chromatid breaks, chromatid exchanges, intra-chromatid deletions, triradials, chromosome breaks, dicentrics, rings, minutes and RB complexes were scored. In each treatment schedule, a minimum of 600 well spread, non-overlapping metaphase plates were scored.

### Evaluation of Mitotic Index (MI)

Mitotic Index was analysed in order to understand the effect of BR on cell proliferation in different treatment schedules. To analyze the rate of mitosis, the numbers of mitotic cells in a total of at least 2000 cells were scored for each treatment schedule. The Mitotic index was calculated by using the formula below.

(1) %MI=NumberofcellsinmitosisTotalnumberofcellsscored×100

### Base excision repair assay

Approximately 1 g of liver from control, Rasayana 1 and Rasayana 2 groups of mice was homogenized with PBS (pH = 7.4, 10 mL) immediately after the sacrifice to obtain a 10% homogenate. The homogenate was then centrifuged at 3000 rpm for 10 minutes at 4°C to obtain a pellet of cells. Whole cell protein extract was prepared by following standard method. Protein content was quantified by Bradford’s method [12]. The required concentration of protein was mixed with Cy3-labeled DNA duplex with one gap. The reaction mix was incubated for 90 minutes at 37°C. After incubation, formamide (20 μL) was added to the sample to stop the reaction and the tubes were kept in boiling water (90°C) for 10 minutes. Tubes were snap cooled in ice for 2 minutes after which the sample and dye were loaded on to 7 M urea gel. The gel was run for 2 hours at 250 V and scanned with Phosphor-imager and analyzed.

### Antioxidant analyses

Rasayanas are potent antioxidants which may have a role in DNA repair. In this regard, BR was also evaluated for its antioxidant activities in mouse test system. The different antioxidant enzymes such as Superoxide dismutase (SOD) [13], Catalase [14], Glutathione S transferase (GST) [15] and GSH [16] were analyzed using the liver tissue of the treated and control animals.

### Reproductive toxicity

Reproductive toxicity was analyzed by sperm count and sperm shape abnormalities. Sperm counts were carried out by following the method of Searle and Beechey [17]. Paired capita were macerated in 1–2 mL of 1% (w/v) trisodium citrate and the resulting suspension was further diluted to 4 mL. After mixing, the spermatozoa were counted using an improved Neubauer haemocytometer. The suspension was mixed with 1% Eosin Y in a 10:1 ratio for the analysis of sperm shape abnormalities [9]. The mixture was allowed to stand for 30 minutes and slides were prepared. These slides were air dried and examined by microscopy for the presence of various sperm shape abnormalities. A minimum of 2000 sperms were counted per animal.

### Statistical analysis

The data are expressed as mean ± SEM. Statistical significance was assessed using Student’s t test. Differences were considered as statistically significant at p <0.05.

## Results

**Figure 1:**
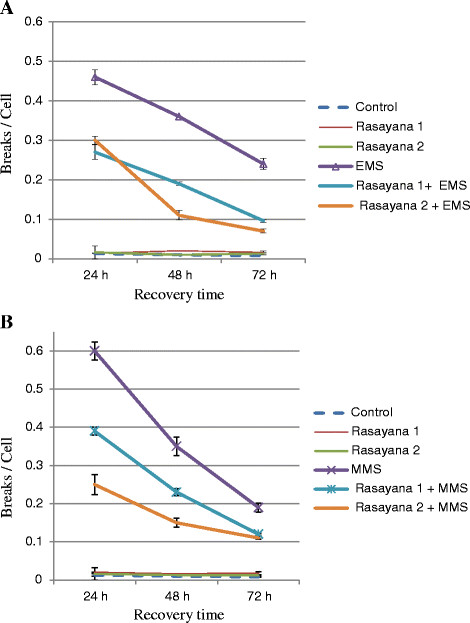
** Chromosome breaks per cell in rasayana and A) EMS- **B). MMS-treated mouse bone marrow cells at different recovery times. EMS or MMS alone (positive controls) elicited high frequencies of breaks per cells whereas the animals without any treatment (negative controls) showed very less frequencies of breaks per cells. Rasayana 1 and 2 produced insignificant (p >0.05) breaks per cell compared to controls. The combined treatments of Rasayana1 and EMS; Rasayana 2 and EMS or Rasayana 1 and MMS; Rasayana 2 and MMS showed significant (p <0.05) reduction in the frequencies of breaks per cell at 24 h, 48 h and 72 h recovery times.

The present study addressed the effect of BR on DNA repair and EMS or MMS-induced chromosomal aberrations in *in vivo* mouse test system. The results obtained after BR treatment are presented in Figure 1 where it is evident that BR treatment alone did not induce significant chromosomal aberrations when compared to that of the control animals (p >0.05). The chromosomal aberrations induced by ethyl methanesulfonate (EMS; 240 mg / kg body weight; positive control) were significant (p <0.05) to that of the control group (Figure 1A).The frequency of breaks per cell in the EMS treated group at 24 h was 0.46 ± 0.019, which was significantly higher when compared to that of total number of breaks per cell in the control group (0.013 ± 0.003) (p <0.05). Similar results were also observed at 48 h (0.36 ± 0.001) and 72 h (0. 24 ± 0.014) which were significantly higher than the control and BR only treated groups (Figure 1A). The frequencies of chromosomal aberrations induced by methyl methanesulfonate (MMS; positive control) was also significantly higher when compared to controls at all recovery times tested (Figure 1B). The chromosomal aberrations induced by EMS and MMS are mainly chromatid breaks, exchanges, intra chromatid deletions and minutes at all the recovery times tested.

Our results also showed that in the BR and EMS combined treatment group, the frequency of chromosomal aberrations was significantly lower (0.27 ± 0.018 for rasayana 1 and 0.30 ± 0.01 for rasayana 2) in comparison to those observed for the EMS only treated group at 24 h. Similar results were observed at 48 h and 72 h respectively (Figure 1A). On par with this, BR and MMS combined treatments also showed a significant reduction in the frequency of chromosomal aberrations at all the recovery times tested (Figure 1B). This is the first of this kind to our knowledge where BR is observed to reduce the clastogenic effects of two well-known alkylating agents viz., EMS and MMS.

Data of mitotic analysis showed that there was reduced number of cells in mitosis in EMS-and MMS-treated groups at 24, 48 and 72 h (Table 1). On the other hand, there was an increase in the number of mitotic cells in animals treated with Rasayana 1 (human equivalent dose calculated based on body surface area) at 24 h. The number of mitotic cells in all the other treatment groups was almost similar to that of controls indicating there is no mitotic selection.

**Table 1:** Mitotic Indices in mouse bone marrow cells of Brahmarasayana and EMS- or MMS- treated groups at different recovery times

**Treatment Groups**	**24 h**	**48 h**	**72 h**
Control	4.70 ± 0.06	4.50 ± 0.28	4.22 ± 0.23
Rasayana I (BSA)	7.40 ± 1.42	4.53 ± 0.63	5.66 ± 0.89
Rasayana II (B.W.)	4.20 ± 0.30	4.06 ± 0.37	4.10 ± 0.05
EMS	2.10 ± 0.17*	2.46 ± 0.08*	2.05 ± 0.12*
Rasayana I +EMS	4.52 ± 0.11	4.03 ± 0.12	4.06 ± 0.14
Rasayana II +EMS	3.06 ± 0.35	3.06 ± 0.08	3.23 ± 0.20
MMS	3.50 ± 0.06	3.54 ± 0.15	3.74 ± 0.15
Rasayana I+ MMS	5.52 ± 0.19	5.62 ± 0.15	5.40 ± 0.15
Rasayana II + MMS	4.62 ± 0.10	4.01 ± 0.11	5.20 ± 0.12

*Significant when compared to control animals (p <0.05).

Reproductive toxicity of BR was evaluated by employing Sperm count and Sperm abnormality assays. The results have shown that there was no variation in the number of sperms in rasayana treated animals when compared to control animals (Table 2). In addition, the analyzed sperm shape abnormalities were also found to be insignificant to that of sperm shape abnormalities in the control group indicating that BR is not inducing reproductive toxicity in the mice (Table 2).

**Table 2:** Sperm count and Sperm abnormalities in Brahmarasayana-treated animals

**Treatment Groups**	**Sperm Count per caput epididymis (million)**	**Total percentage of Sperm Abnormalities**
Control	4.40 ± 0.46	2.57 ± 0.45
Rasayana I (BSA)	4.85 ± 0.29	2.60 ± 0.25
Rasayana II (B.W.)	3.62 ± 0. 62	2.05 ± 0.07

**Figure 2:**
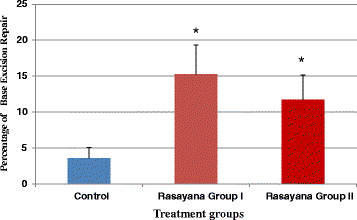
** Constitutive base excision repair in BR-treated animals after 24 h recovery time.** Constitutive repair in Rasayana 1 and Rasayana 2 treated animals are significantly (p <0.05) higher compared to control animals

Constitutive base excision repair capacity was tested in BR-treated and control animals by employing cell free liver extracts. It is interesting to note that there was increased repair in BR-treated animals when compared to controls (Figure 2) indicating enhancement of DNA base excision repair.

Rasayanas are potent antioxidants which may have a role in DNA repair. In this direction, BR was also evaluated for its antioxidant activities. The results have revealed that there was marginal elevation in the SOD level in Rasayana 1 and EMS-treated animals when compared to the controls. The groups receiving BR and EMS combination treatments showed maximum SOD activity when compared to all other treated groups of animals suggesting antioxidant activity of BR (Table 3). Catalase activities were reduced in combined treatment of BR and EMS when compared to EMS and controls. However in case of MMS treatment, combined treatment of rasayana 1 and MMS showed higher levels of catalase activity in comparison to MMS only. This may be the result of differential action of EMS and MMS. The data also indicate that in the Rasayana 2, EMS- and MMS-treated animals show little GST activity when compared to other treatment regimens. On the other hand, there was no significant variation in GSH levels in all the treatment groups (Table 3).

**Table 3:** Anti-oxidant levels in BR- and EMS- or MMS- treated animals

**Treatment Group**	**SOD Activity / mg protein**	**Catalase Activity / mg protein**	**GSH activity / mg protein**	**GST activity / mg protein**
**Control**	0.02 ± 0.01	5.32 ± 1.09	2.44 ± 0.53	8.26 ± 1.32
**Rasayana I**	0.03 ± 0.01	7.69 ± 2.85	2.15 ± 0.28	7.11 ± 2.68**
**Rasayana II**	0.01 ± 0.01	5.13 ±0.13	2.66 ± 0.02	1.46 ± 0.09**
**EMS only**	0.03 ± 0.01	10.73 ± 2.31**	1.55 ± 0.79**	2.09 ± 0.90**
**Rasayana I + EMS**	0.04 ± 0.01*	4.09 ± 0.55	2.75 ± 0.00	6.94 ± 2.54
**Rasayana II + EMS**	0.05 ± 0.01*	4.59 ± 0.16	2.48 ± 0.09	11.47 ± 6.36
**MMS Only**	0.02 ± 0.01	2.68 ± 1.16**	2.83 ± 0.07	0.72 ± 0.18**
**Rasayana I + MMS**	0.05 ± 0.00*	6.17 ± 1.29*	1.81 ± 0.66	8.92 ± 5.17
**Rasayana II + MMS**	0.04 ± 0.01*	3.80 ± 1.23	2.48 ± 0.28	9.91 ± 2.44

* Significant when compared to animals treated with EMS or MMS only (p <0.05).

**Significant when compared to Control animals (p <0.05).

## Discussion

It is beyond doubt that DNA is the primary target of several chemicals and physical agents which cause the genetic alterations viz. point mutations, chromosomal aberrations, sister chromatid exchanges (SCEs) and others. These induced alterations in the DNA, if accumulated leads to various health hazards including cancer. However, there are several mechanisms to repair the damaged DNA in the living organisms, which are responsible to maintain the stability of genetic material. Defective or inefficient repair machinery may not combat efficiently the genotoxic effects induced by the agents. Furthermore, the efficiency of DNA repair machinery may also be comparable to rate of repair / removal of DNA damages. Therefore, we hypothesize that the DNA repair efficiency may be modulated by rasayanas. Hence, in the present investigation, an attempt was made to understand the effect of Brahmarasayana, a well-known Medhya rasayana on EMS- and MMS- induced chromosomal aberrations / repair of chromosomal aberrations in an *in vivo* mice system.

Two different doses of BR, one based on body surface area extrapolated from the Human Equivalent Dose (HED) [18] and another based on the body weight of animals (which is equivalent to the human dose administered by Ayurveda doctors to the patients) were employed to overcome the discrepancies with respect to several parameters in mammalian species [18]. Further, in Ayurveda rasayanas are generally administrated for longer duration. Hence, in the present study, animals were treated for 60 days. The results of the present studies have clearly shown that the treatment of BR, elicited insignificant (p >0.05) number of breaks per cell when compared to that of breaks observed in control animals, as observed in the earlier study [9], indicating no genotoxicity of BR. On the other hand, significantly higher frequencies (p <0.05) of chromosomal aberrations were induced by positive controls such as EMS and MMS. Furthermore, it was observed that the chromosomal aberrations induced by EMS and MMS were mainly chromatid breaks, exchanges, intrachromatid deletions and minutes at all the time points tested. These results were similar to the earlier observations [10,19-22]. EMS and MMS are both alkylating agents, electrophilic compounds, with the ability to attack the nucleophilic centres of DNA and capable of inducing a variety of lesions including DNA adducts, cross-links and strand breaks, which can be expressed as chromosomal aberrations. It is interesting to note that there was significant (p <0.05) reduction in the frequency of chromosomal aberrations induced by EMS or MMS in animals pre-treated with BR (Figure 1) indicating either prevention of EMS induced chromosomal damage or enhancement of DNA repair by BR. The differences between MMS and EMS-induced breaks can be explained either by differences in the patterns of alkylation by the two compounds (e.g., the amount of N3-alkyladenine versus the amount of O^6^-alkylguanine) or by differences in the way in which cells process the respective adduct(s). There are different mechanisms which lead to chromosomal aberrations such as mis-repairing of double strand breaks [23], stickiness and direct breakage of phosphor di-ester bonds of DNA by alkylating agents [24] and others which rely on DNA damaging agents; and free radical generation and DNA damage is one such mechanisms. The future work needs to be focused on unravelling the mechanistic pattern of cellular damage with respect to free radicals and alkylating agent-induced chromosomal damage. Furthermore, this is the first of this kind to our knowledge where BR is reducing clastogenic effects of two well-known alkylating agents viz., EMS and MMS. Consistent with this, Sharma *et al.*[25] have shown that *Emblica officinalis* plant extract, one of BR’s principal ingredients, has an inhibitory effect on mutagen-induced chromosomal anomalies in Swiss albino mice. Oral administration of plant extract (250 and 500 mg/kg body weight) for 7 days together with single doses of the mutagens, Benzo (a) pyrene (125 mg/kg body weight) or cyclophosphamide (40 mg/kg body weight intraperitonially), significantly reduced frequencies of chromosomal aberrations and micronuclei in Swiss albino mice were observed compared to treatment with mutagens alone. Administration of rasayana namely *Chyavanaprasha* (5, 7.5,10,15, 20, 40 and 80 mg/kg body weight) for 5 consecutive days and treatment later with 10 Gy of gamma- rays also showed an alleviation in symptoms of radiation sickness like reduction in food and water intake, irritability, weight loss, emaciation, lethargy, diarrhea and facial edema. There was also a delay in mortality of animals indicating radioprotective activity of *Chyavanaprasha*[26]. Another rasayana viz., *Triphala* also showed radioprotection in mice [27]. The *in vivo* treatment with *Triphala* (oral: 40 mg/ kg body weight) on mice transplanted with mouse thymic lymphoma (barcl-95) cells produced significant reduction in tumor growth. *Triphala* was shown to induce cytotoxicity in tumor cells but not in normal cells and appears to be related to its ability to evoke differential response in intracellular ROS generation [28]. Similar anti mutagenic activity of *Triphala* was observed in 115 (S115) and MCF-7 breast cancer cells as well as PC-3 and DU-145 prostate cancer cells [29]. Joshi and Parle [30] have treated mice with different concentrations of *Brahmi rasayana* and demonstrated that the high dose of *Brahmi rasayana* (200 mg/kg) elicited improved memory and learning in mice when compared to mice treated with scopolamine. Further, pre-treatment with *Brahmi rasayana* for 8 days protected both young and old mice against scopolamine-induced amnesia.

In the present investigations, constitutive base excision repair capacity was also tested in BR treated and control animals in support of protective activity of BR, by employing cell free liver extracts. It is interesting to note that there was increased repair in BR treated animals in comparison to controls (Figure 2) indicating enhancement of DNA base excision repair. This may be related to the increased ability of the repair machinery or amount of the enzymes in the cells, which needs to be addressed in future. We did not assess the BER capacity in EMS and MMS-challenged animals or animals treated with BR and EMS or MMS-combined treatments as they already possess inherently high levels of DNA damage and repair and it is difficult to discern differences in BR-treated and clastogen only-treated groups.

Data of Mitotic analysis showed that there was reduced number of cells in mitosis in EMS and MMS-treated groups at 24, 48 and 72 h (Table 1). This is understandable as cells with a large number of breaks stop cell cycle. However, there was an increase in the number of mitotic cells in animals treated with Rasayana 1 (human equivalent dose calculated based on body surface area) at 24 h whilst the number of mitotic cells in all the other treatment groups was almost similar to that of controls indicating there is no mitotic selection. This particular finding validates the use of two dosing regimens. We find that *Rasayana 1*, in addition to being more protective, also induces greater mitotic cellularity. Utilizing human equivalent doses (as in *Rasayana 1*) of Ayurvedic formulations for animal studies present a more realistic outcome in terms of translation and by this notion BR elicits better protection, at least in the mice treated with *Rasayana 1*. On a par, BR treated irradiated mice, showed increase in total leucocyte count and bone-marrow cellularity [31]. Kumar *et al*[32] have reported the immuno-stimulatory property of BR in mice. It enhanced proliferation of spleen cells, increased the activity of esterase and humoral immune response. Administration of BR to cancer patients undergoing radiotherapy also resulted in the recovery of haematopoetic system, as shown by increased levels of neutrophils and lymphocytes, reduction of leucopenia, neutropenia and lymphopenia [33]. Furthermore, cancer chemotherapy with cyclophosphamide results in myeolosuppression and BR found to reduce myelosuppression and subsequent leucopenia [34].

Reproductive toxicity of BR was analyzed by employing Sperm count and Sperm abnormality assays. The results have shown that there was no variation in the number of sperms in BR-treated animals when compared to control animals (Table 2). In addition the analyzed sperm shape abnormalities were also found to be insignificant to that of sperm shape abnormalities in control animals indicating BR is not inducing reproductive toxicity in the mice (Table 2). These results are in accordance with our earlier findings [9].

Rasayanas are potent antioxidants which may have a role in DNA repair. In this regard, BR was also evaluated for its antioxidant activities in mouse test system. Assays evaluating the SOD, catalase, GST and GSH activities in the control and other experimental groups have revealed marginal differences as indicated in Table 3. Whilst cited studies do reveal antioxidant potential of rasayana, the assessment through the various enzymes could also be organ specific. Singh *et al.*[35] evaluated the plant *Tinospora cordifolia* against oxidative stress on Swiss albino mice and found that SOD, catalase and GST levels were significantly increased in the lung whilst in the kidney, only catalase and SOD were increased at both dose levels of treatment given. However, Rekha* et al*[4,36] have shown that BR treatment resulted in increased levels of SOD and catalase in irradiated mice. Rekha* et al*[37] also examined free oxygen radical scavenging activity of BR in both *in vivo* and *in vitro* test systems and found that the lipid peroxides, hydroxyl radicals, superoxides, nitric oxide and nitrite production were reduced by BR indicating antioxidant effect of BR. Male chickens treated with BR (2 g/ kg body weight) for 10 days and heat stressed (40 ± 1°C for 5 or 10 days) showed enhanced action of enzymatic and non-enzymatic antioxidants and also nullified the effects of free radicals generated during heat stress. Further, they observed significant increase in the haemoglobin content in these BR treated and heat-stressed birds compared to birds subjected to heat-stress alone. In addition the lipid peroxidation level was significantly higher in heat stressed birds. However the administration of BR to the stressed animal showed reduction in the lipid peroxidation and this was brought back to an almost insignificant level, in contrast to that observed in control set of birds [5]. Furthermore, the mice (C3H/HeN) when administered with MAK 5 (Maharishi Amrit Kalash 5), another rasayana formulation at concentrations of 50 mg/kg, 100 mg/kg or 200 mg/kg daily per day (3 days /week) for 2 months showed significant increase in glucose consumption by peritoneal macrophages. Further, the production of IFN-gamma and IL-2 was significantly higher in the rasayana-treated group of animals when compared to the control group of old mice suggesting that MAK 5 suppressed the age-associated glucose consumption of peritoneal macrophages and cellular immune function reduction which, in turn, prevented the immune senescence [38].

## Conclusion

The study indicates that BR reduced EMS- and MMS-induced chromosomal aberrations and enhanced the constitutive base excision repair capacity in mice test system. The study provides *in vivo* evidence in mice for the anticlastogenic activity of BR and hence a potential for further evaluation in humans on the protective effects of such formulations in cancer treatments where patients receive high doses of radiation and chemotherapy and in anti-ageing research. The components of Rasayana contribute immensely to the polyherbal’s observed biological effects. The array of phytochemicals present within the two major ingredients of BR include kaempferol, phyllembic acid, corilagin, ellagic acid, linoleic acid, pyrogallol, betulinic acid, chebulagic acids and oleic acid, amongst others [39-43] and may be responsible for the activities observed in this study. The two major constitutents, *Phyllanthus emblica* and *Terminalia chebula* have individually shown significant antioxidant activity and preparations containing these two plants, such as Triphala have shown the same [44]. However the antioxidant potential observed in this study, using liver tissue, differed from published reports and may reflect the organ specificity with respect to free radical scavenging potential, as highlighted above. The study is limited at present to evidence generated from studies on mice and further exploration is therefore required to establish if similar effects are seen in humans. Further investigations related to the molecular mechanisms involved in the enhancement of repair are warranted.

## Competing interests

The authors declare that they have no competing interests.

## Authors’ contributions

KPG, AS, VJS, RSKS, carried out the study. KPG wrote the manuscript. VS contributed to the manuscript corrections and editing in addition to valued suggestions. PMV supplied BR and suggested to use the same to carry out experiments to understand its beneficial role. KS and PMG supervised the work and the manuscript writing. All authors read and approved the final manuscript.

## Pre-publication history

The pre-publication history for this paper can be accessed here:

http://www.biomedcentral.com/1472-6882/12/113/prepub
